# Inherited Disease Genetics Improves the Identification of Cancer-Associated Genes

**DOI:** 10.1371/journal.pgen.1006081

**Published:** 2016-06-15

**Authors:** Boyang Zhao, Justin R. Pritchard

**Affiliations:** 1 Computational and Systems Biology, Massachusetts Institute of Technology, Cambridge, Massachusetts, United States of America; 2 Discovery/Translational Biology, ARIAD Pharmaceuticals, Cambridge, Massachusetts, United States of America; HudsonAlpha Institute for Biotechnology, UNITED STATES

## Abstract

The identification of biologically significant variants in cancer genomes is critical to therapeutic discovery, but it is limited by the statistical power needed to discern driver from passenger. Independent biological data can be used to filter cancer exomes and increase statistical power. Large genetic databases for inherited diseases are uniquely suited to this task because they contain specific amino acid alterations with known pathogenicity and molecular mechanisms. However, no rigorous method to overlay this information onto the cancer exome exists. Here, we present a computational methodology that overlays any variant database onto the somatic mutations in all cancer exomes. We validate the computation experimentally and identify novel associations in a re-analysis of 7362 cancer exomes. This analysis identified activating SOS1 mutations associated with Noonan syndrome as significantly altered in melanoma and the first kinase-activating mutations in *ACVR1* associated with adult tumors. Beyond a filter, significant variants found in both rare cancers and rare inherited diseases increase the unmet medical need for therapeutics that target these variants and may bootstrap drug discovery efforts in orphan indications.

## Author Summary

Current identification of driver cancer genes is limited by the statistical resolution of existing approaches. However, many rare mutations that contribute to cancer progression remain unidentified. We capitalize on the powerful insight that our knowledge of genetic variants in inherited diseases can enhance driver oncogene identification. Inherited disease variants are unique because they have known pathogenicity, validated causality, and resolution at the level of individual amino acids. Because of this, it is commonly argued, that overlapping mutations between a given inherited disease dataset and human cancer are important by virtue of the fact that they occur in both places. However, this argument never takes into account how often a discovered mutant would overlap with the cancer exome by chance. We find that chance overlap of variants happens surprisingly often. To exclude this effect, we rigorously estimate how often overlap can occur by chance. Beyond the discovery of new cancer-associated genes, we independently validate our predictions with experiments, structural analysis, and epidemiological data. Finally, our ability to rigorously link drivers across diseases has the potential to create a social benefit by bridging the treatment of rare inherited diseases and the treatment of rare cancers. This holds the potential to enhance patient access to new medicines by making orphan diseases a little more common and identifying larger unmet medical needs.

## Introduction

Statistical approaches to identify new cancer drivers have revolutionized our view of the cancer genome [1–4]. However, state-of-the-art statistical approaches still fail to identify bona fide cancer drivers. For instance, activating kinase mutations in *FGFR2* and *FGFR3* in lung squamous cell carcinomas were overlooked in previous computational and statistical analyses because the genomic cohort sizes were insufficient [3] to accurately predict their significance [5].

While increasing cohort sizes is a conceptually simple (but costly) way to increase the statistical power to detect rare variants in cancer genomes, an exciting alternative is to systematically overlay independent biological information [6,7]. Inherited disease databases harbor useful biological information that includes: resolution at the level of specific amino acid residues, documented modes of inheritance, and even molecular mechanisms. Thus, while the joint information contained in inherited diseases and cancer databases is widely believed to be useful [5,8–10], there is not a statistically rigorous methodology for validating the finding that a variant exists in both databases, while properly excluding false-positive connections. In fact, the observation of overlap between variants in a genetic disease and cancer is often cited as important without any idea of how often raw overlap would occur by chance. And while germline diseases associated with cancer susceptibility are of obvious relevance, a method is urgently needed that can systematically leverage the power of the two datasets of variants to find genetic drivers in noisy genomics data. Rigorously identifying variant overlap between inherited diseases and sporadic cancers will create larger patient populations by stitching together rare cancer subtypes and inherited diseases that have similar underlying molecular alterations. Discovering a larger population of diseases harboring a particular mutation is a novel method to speed drug discovery by identifying a larger population with unmet medical needs.

Importantly, beyond this particular application of inherited disease databases, there is also a broader need for a statistical method that can discern whether a variant or set of variants that is identified in any study is significantly altered in human cancer. Biophysical, biochemical, and saturating mutagenesis studies are an incomplete list of approaches that generate functional data at the amino acid residue level. Determining whether these functional studies have identified positions in proteins that are significantly altered in human cancer is a critical step in understanding their clinical significance.

## Methods

### Datasets

We acquired the following datasets: cancer mutations (mutation annotation format [MAF] files, downloaded via cBioPortal [11] application programming interface [API], August 23, 2014), UniProt HUMSAVAR [12] (release 2014_07 of July 9, 2014), ClinVar [13] (file date 20140807), RNA-Seq V2 RSEM data (upper quartile normalized, downloaded via cBioPortal API, October 24, 2014), clinical data (downloaded via cBioPortal API, December 17, 2014), UniProt human proteome (downloaded from UniProt, September 11, 2014), and HUGO IDs (Human Genome Organization identifications; downloaded from the HGNC [HUGO Gene Nomenclature Committee] website, September 18, 2014).

The UniProt HUMSAVAR dataset was filtered to remove silent mutations and to only consider mutations with pathogenic consequences. This resulted in a subset of the HUMSAVAR data set that contains known pathogenic disorders. No polymorphic alleles with questionable disease relevance were retained. Silent mutations that cause splicing defects were retained. By comparing across the HUMSAVAR and cancer datasets, we removed any entries with discrepancies in the reference amino acid residues. All overlapping pathogenic variants were hand annotated by their mechanism of pathogeneicity (i.e autosomal dominant/recessive etc). We also acquired RNA-Seq expression data for all the genes in the cancer mutations dataset. For any of the few TCGA entries for which the RNA-Seq data were not available, we used the median of the available expression values for given gene and given tumor type. Expression data were used for dataset stratification of expressed versus nonexpressed genes per tumor type and in determining statistical significance cutoffs, as described in later sections. We also acquired the UniProt human proteome (and used HGNC Hugo IDs for ID mapping) to determine the protein length of the canonical protein for each gene. This information was later used in our statistical model for estimating mutation burden.

It is important to note that exome capture methods and variant calling pipelines are different across cancer studies. Furthermore, distinct TCGA working groups curate their data to tune sensitivity and specificity. Thus, across all studies, the sensitivity of the given exome analysis pipeline to call a particular variant can vary. Because we do not compare mutational data across cancer types and studies, the differences in curation will not lead to incorrect comparisons. All of our simulation based statistics are based upon individual studies. Importantly other pan-cancer analysis efforts also utilize curated data [1,14]. However, a detailed analysis of the sensitivity of our algorithm to these differences is presented in S1 Text and S1 Data as well as S4 and S5 Figs. Hit lists for different inputs are in our GitHub repository under the siglist folder.

Different inherited disease variant databases draw and curate from different data sources. Please refer to the supplemental results and discussion for a detailed comparison between HUMSAVAR and ClinVar.

### Computational model

**Fig 1:**
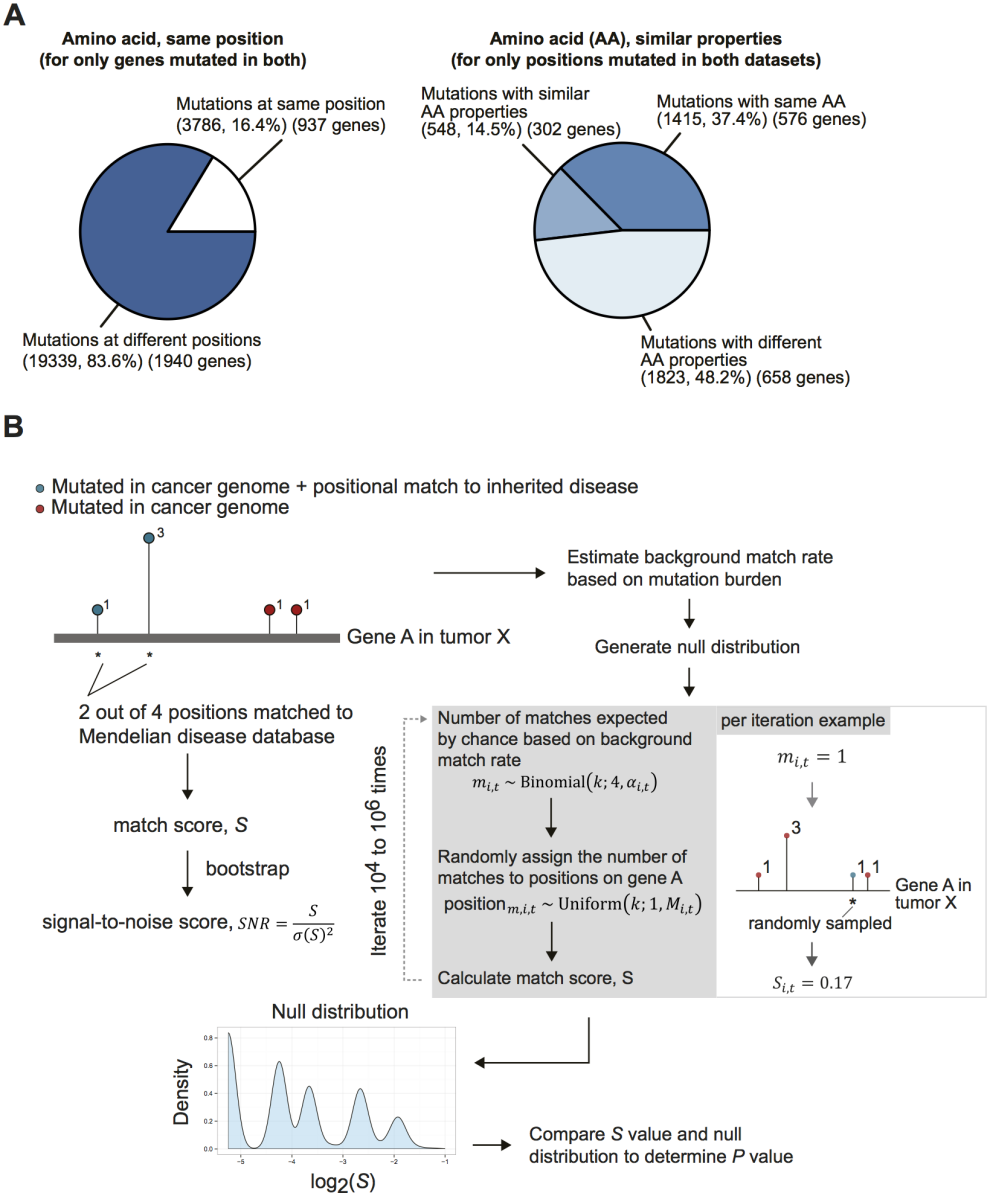
Summary statistics of overlay between inherited diseases and cancer mutation datasets and computational model overview. **(A)** Breakdown of the shared mutations between the two datasets, showing the percentage with an exact positional match, and of those which are mutations with the exact same or similar residue changes. **(B)** Refer to Methods for details of methodology. We derived a match score based on the overlay between the inherited diseases and cancer mutation datasets. A signal-to-noise ratio (SNR) was derived through bootstrapping (*N* = 1000)—sampling with replacement from all of the available reported cases of cancer mutations for a given gene in a given tumor type with recalculations of the match score value. A *P* value also was derived for the match score using the empirical null distribution, which was generated through a permutation procedure. In each iteration, the number of matches expected by chance was determined based on a binomial distribution (with probability equal to the estimated background match rate). For the number of matches determined, they are randomly assigned based on a uniform distribution to one of the available mutated positions. A match score was subsequently calculated. This process was repeated 10^4^–10^6^ times to generate the empirical null distribution for the match score.

Our approach involved the development of a match score (per gene per study) to make comparisons between the inherited diseases dataset and the cancer genome dataset (Fig 1A and 1B). We used a bootstrapping approach to assess the confidence in the score, by deriving a signal-to-noise ratio. We also have used a permutation-based approach to determine the empirical null distribution and calculate the *P* value for each gene (Fig 1C). The algorithm implementation and subsequent analyses were written in R.

A match score (*S*) for given gene *i* in tumor type *t* was defined as the proportion of observed cases (out of the total observed cases for a given gene in the cancer dataset of tumor type *t*) reported at residue positions that are matched to inherited diseases (based on one of the match criteria).

$$S_{i,t}=\frac{\#\left\{\text{matched}\;\text{cases}\;\text{for}\;\text{gene}\;i,\;\text{tumor}\;\text{type}\;t\right\}}{\#\left\{\text{total}\;\text{cases}\;\text{for}\;\text{gene}\;i,\;\text{tumor}\;\text{type}\;t\right\}}$$ (1)

To assess the confidence we have for each mutation, we performed bootstrapping (*N* = 1000) from the available reported cases for a given gene and tumor type to calculate a standard deviation of the score and, subsequently, a signal-to-noise ratio (*SNR*):

$$SNR_{i,t}=\frac{S_{i,t}}{\sigma {\left(S_{i,t}\right)}^{2}}$$ (2)

The null distribution (*ρ*_*i*,*t*_) for gene *i* in tumor type *t* is a convolution of the distributions of score (*ψ*_*k*_) calculated based on a background match rate across all possible numbers of matches (*k*). The probability of the match *k* is calculated based on a binomial distribution:

$$\rho_{\text{i},\text{t}}\;\sim \sum_{\text{k}=0}^{{\text{n}}_{\text{i},\text{t}}}\psi_{\text{k}}\text{Bin}\left(\text{k};{\text{n}}_{\text{i},\text{t}},\alpha_{\text{i},\text{t}}\right)\;$$ (3)

We defined the match rate as the probability of a match between the cancer mutation dataset (per tumor type) and the inherited diseases dataset given an observed mutation in a given gene. We assumed that the match rate (*α*_*i*,*t*_) for gene *i* in tumor type *t* is a function of the mutation burden:

$$\alpha_{i,t}=f\left(\mu_{i,t}\right)$$ (4)

Here we used a linear model to approximate the match rate,

$$\alpha_{i,t}=\gamma_{t}\mu_{i,t}$$ (5)

where *γ*_*t*_ is a tumor type—specific proportionality coefficient.

We estimated mutation burden as the number of unique positions mutated *n* over the protein length *L*:

$${\hat{\mu }}_{i,t}=\frac{n_{i,t}}{L_{i,t}}$$ (6)

We estimated a background proportionality coefficient as the mean of coefficient values from the entire dataset (with *N* genes),

$${\hat{\gamma }}_{t}=\frac{1}{N}\underset{i,\;t}{\sum^{\;}}\frac{{\hat{\mu }}_{i,t}}{{\hat{\alpha }}_{i,t}}$$ (7)

where the estimated match rate ${\hat{\alpha }}_{i,t}$ for gene *i* in tumor type *t* is the ratio of the number of unique positions mutated and matched between the two datasets *m* and the number of unique positions mutated *n*,

$${\hat{\alpha }}_{i,t}=\frac{m_{i,t}}{n_{i,t}}$$ (8)

Here we tried to estimate the background proportionality coefficient with various expression level cutoffs and observed the estimation to be stable (S1 Fig).

In practice, the empirical null distribution was generated using a permutation procedure. Each mutation was sampled as either a match or a nonmatch based on a background match rate probability (${\hat{\alpha }}_{i,t}={\hat{\gamma }}_{t}{\hat{\mu }}_{i,t}$) as a Bernoulli process. Then for each mutation that is a match, the actual residue position mutated was determined based on a uniform distribution (from one of the mutated positions in the cancer dataset for a given gene in a given study). The match score (*S*_*i*,*t*_) was then calculated for that iteration. This was sampled for a minimum of 10^4^ times and a maximum of 10^6^ times to generate the empirical null distribution of *S* for a given gene, and a *P* value was calculated thereafter. The nominal *P* values were corrected for multiple hypotheses using the Benjamini-Hochberg False Discovery Rate method, yielding an adjusted *P* value for each gene. We determined the adjusted *P* value cutoff based on the first quartile—adjusted *P* value from the nonexpressed genes from pan-cancer data. We assumed here that any statistical significance for nonexpressed genes would constitute false-positives and that the overall cumulative distribution of nominal *P* values of expressed genes would have a much longer tail, with smaller *P* values and adjusted *P* values for the significantly mutated genes. It is conceivable that a variant in a nonexpressed gene could have an important role, i.e a regulatory function. However, we still consider nonexpressed genes to constitute a “false positive” distribution, and suggest that at worst this may make thresholding against it a little conservative. We generated empirical cumulative distribution functions for expressed and nonexpressed genes and consistently observed a difference in distributions (S2A Fig). We also used RNA-Seq cutoffs of one, five, and ten reads and the results were stable (S2B Fig).

#### Code and data accessibility

All input files, R code, and associated documentation have been deposited on GitHub https://github.com/boyangzhao/targetID.

#### Simulated data generation/analyses

We tested the robustness of the approach using simulated data by analyzing a randomly generated synthetic dataset over a range of parameter values. Specifically, we randomly generated 1000 simulated genes with the following parameters: protein length (10–8000 amino acids), number of exact positional matches (1–100 unit matches), match score (0.01–0.99), background mutation burden (from a single unique nonmatched positional mutation to the maximal possible number of unique nonmatched positional mutations), and total number of cases (scaled by a factor between 1 and 50). Unit measurements were defined as the smallest number of cases needed to satisfy the given match score.

#### Analysis of datasets

Significant hits were filtered to contain at least two reported cases, a signal-to-noise ratio of greater than 2, and an adjusted *P* value cutoff based on the first quartile—adjusted *P* value from the pan-cancer nonexpressed genes.

#### Clinical data

The Biotab data was downloaded from the TCGA data portal on 01/08/16. Relevant clinical variables used for analysis included the breslow thickness at diagnosis, clark level at diagnosis, tumor staging by AJCC criteria, and the site of the sample collected by TCGA.

#### Survival analyses

Survival analyses were performed using the standard Cox proportional hazards model (implemented in R package “survival”). Here, a hazard ratio >1 suggests that the mutation has a worse outcome, whereas a value <1 suggests a better outcome. Age, gender, mutation number and tumor stage were used as covariates. ExaLT, which was specifically designed for unequal sample sizes in TCGA data was compiled and run using the—table command according to the author’s readme on GitHub [15].

#### Epidemiology

All epidemiology was based on a US population estimate of 318 million people. Incidence estimates are per 1-year period. Fibrodysplasia ossificans progressiva (FOP) has a described incidence of 1 in 2 million [16]. Pediatric high-grade glioma has a total incidence of 0.85 per 100000 [17]. The incidence of activin A receptor type 1 (*ACVR1*) mutations in pediatric high-grade gliomas was recently reported at 20–30%[10,18–20]. The pediatric proportion of the US population is estimated to be 30%. Endometrial cancer has an incidence of 25 per 100,000 women according to National Cancer Institute Surveillance, Epidemiology, and End Results Program statistics [21]. We find that *ACVR1* mutations that match FOP are in 1.65% of endometrial cancers and that some type of *ACVR1* mutations are in 3.3% of endometrial cancers.

#### Experiments

Human SOS1 was synthesized without the spe1 restriction site and cloned into PLVX-IRES-PURO. Point mutants were made by site-directed mutagenesis. HEK293T cells (American Type Culture Collection) were seeded to 60–80% confluency and transfected with Lipofectamine 2000 in 24- and 96-well plates. Thirty-six hours after transfection, cells were serum starved for 4–8 hours and evaluated for phospho-ERK 1/2 and total-ERK1/2 ELISA kits from Cell Signaling Technologies. A positive control lysate was prepared by stimulating cells with 20-ng/ml EGF from R&D systems for 5 minutes. This lysate was serially diluted 128-fold and fit to a standard curve. A standard curve was run on each ELISA plate to quantify relative amounts of total and phospho-ERK. Phosphorylated ERK measurements were then corrected for the total ERK measurement, and quantified relative to the SOS1 wild-type transfection. PLVX-BRAF V600E-IRES-PURO was used as a positive control. The experiment was run in the presence or absence of phosphatase inhibitors in the lysate. The average of N = 8 biological replicates across both experimental lysis conditions (N = 4) is reported. Both lysis conditions were performed separately but analyzed on the same ELISA plate.

## Results and Discussion

### Extensive overlap of variant amino acids and known false-positives suggests the need for a rigorous statistical approach

**Fig 2:**
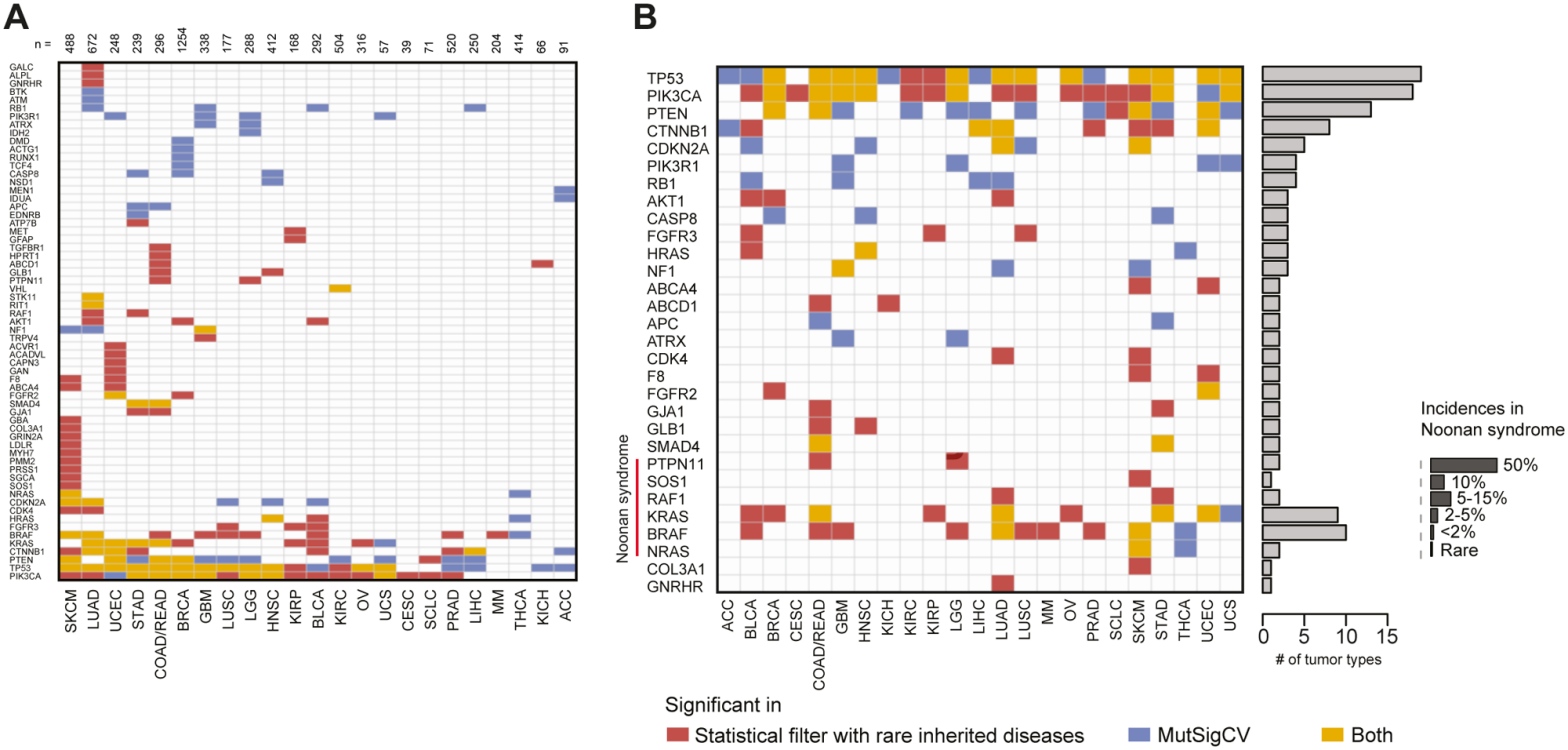
Overall summary of identified mutant variants. **(A)** Statistically significant hits based on a signal-to-noise ratio cutoff of 2, at least two reported cases, and a *P* value cutoff of 0.062 based on the first quartile of adjusted *P* values from the analyses of all exact matches in nonexpressed genes (see methods). The number of input exomes for given tumor type is indicated at the top of the heatmap. **(B)** Highlights of hits with significance in at least two tumor types, genes involved in Ras/mitogen-activated protein kinase pathways and Noonan syndrome.

With the goal of identifying genes that significantly overlap between inherited disease databases and cancer exomes, we merged data from the pathogenic entries in the UniProt HUMSAVAR [12] database and the entire set of tumor exomes featured on the cBioPortal [11]. Raw overlap of the data identified 576 genes that had an exact match between an inherited disease and a cancer exome (i.e., the same amino acid alteration at the same site) (Fig 1A). A statistics-naïve inspection of the most frequent overlapping genes identified false-positives such as the presence of *RYR2* in eight different cancer types (S6B Fig). *RYR2* has been shown to be heavily mutated relative to the whole genome background mutation rate in previous pan-cancer analyses [2]. The existence of a large amount of mutational overlap between the datasets (approximately four times the size of current pan-cancer gene lists) and known false-positives (such as *RYR2*) highlight the need for rigorous statistics that correct for local mutation densities, gene length, cancer type, and gene expression (S6A–S6D Fig). To this end, we developed a suite of statistics for effective analysis (Fig 1B). Briefly, we generate a match score and a corresponding signal-to-noise ratio by bootstrapping. Subsequently, we use a permutation-based approach to create an empirical null distribution of the match score. Our approach eliminated the false-positive *RYR2* from our set of commonly overlapped genes, allowed us to detect *FGFR2* and *FGFR3* as significantly mutated, and identified 49 significantly overlapping genes (S6 Fig and Fig 2A and 2B). Extensive simulations demonstrated that our approach corrects for biases in protein length and mutation density across different cancer types (S7 Fig). We also conservatively reasoned that non-expressed genes should not be considered hits and likely constitute false-positive overlap. We plotted the cumulative distribution of *P* values for expressed and non-expressed genes and observed that expressed genes have a much longer *P* value tail than non-expressed genes (S2 Fig). A long tail for expressed genes relative to a non-expressed “false positive” gene set suggests that our algorithm enriches for information and is indicative of a low false-positive rate. We used the distribution to exclude hits that were not expressed or that failed to be more significant than the non-expressed genes.

A recent paper by Melamed et al [22] attempted to identify driver genes by using patterns of comorbidities between inherited diseases and human cancers. A “top-down” approach, this comorbidity based assessment makes the strong assumption that the patterns in the incidence of common clinical conditions can identify genetic similarities and thus drivers of cancer. Importantly, comparing significantly overlapping individual genes between comorbid cancers and Mendelian diseases yielded only four significant putative drivers in 14 inherited disease—indication pairs, all of which were previously known. While this study performs many other interesting analyses, the power of their direct genetic comparison to inherited disease data is limited. A comparison with our approach demonstrates the power of examining the overlap between inherited diseases and cancer at the level of individual residues (S3 Fig, S1 Text).

### Noonan syndrome genetics adds sensitivity to detect cancer-associated variants that activate the RAS/MAPK pathway

**Fig 3:**
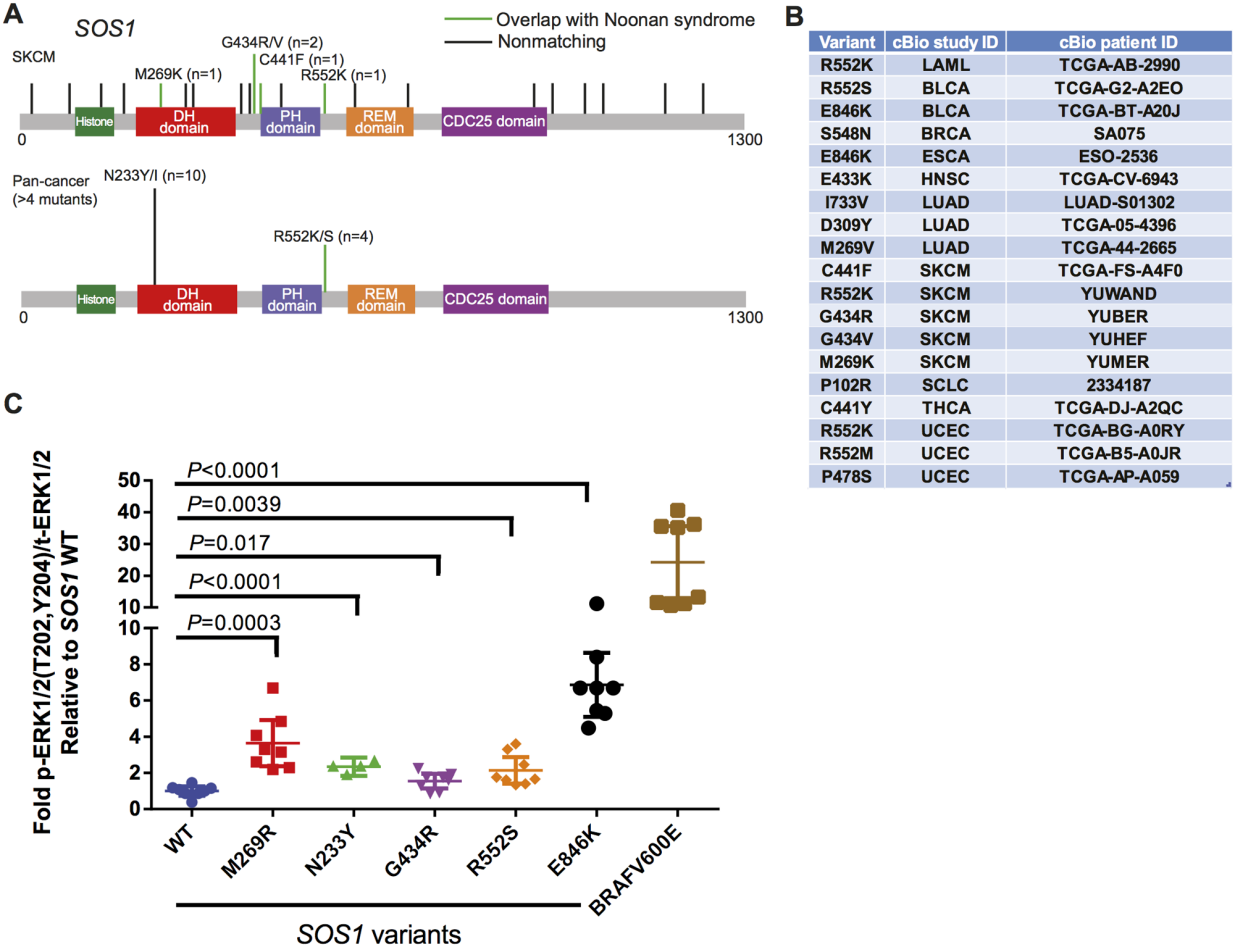
*SOS1* mutations are significantly associated with cancer and activate the RAS/MAPK pathway. **(A)** Top: All four skin cutaneous melanoma datasets in cBioPortal are presented. Vertical lines indicate mutations in *SOS1*. Green lines identify overlap with Mendelian diseases. Bottom: A pan-cancer version of the *SOS1* mutational data is presented. Data is filtered for N ≥ 4. **(B)** A table containing all *SOS1* variants across the TCGA that match a known Noonan syndrome—associated residue. cBioPortal study ID refers to the indication name in the cBioPortal. The patient ID is the unique sample identifier for the given study. **(C)** HEK293T transfection experiment for phosphorylated ERK1/2. Dots indicate biological replicates (N = 8). *P* value was assessed by Student *t* test.

We observed a striking sensitivity to detect the overlap between specific molecular etiologies of the inherited disease Noonan syndrome and rare pan-cancer variants in *PTPN11*, *RAF1*, and *SOS1* (Fig 2B, highlighted in heatmap). We found 26 instances of significant overlap across Noonan syndrome disease genes. We compared these to the hits called by MutSigCV—Broad Institute’s somatic mutation significance caller based on modeling of the background mutation rate, controlled for various covariates, e.g. replication time, transcriptional activity, and chromatin state. MutSigCV identified only 10 hits (of these, seven were in both datasets). While Noonan syndrome genes constitute long-studied positive controls, to the best of our knowledge we provide the first evidence of significantly overrepresented somatic mutations of *SOS1* in a subtype of human cancer(melanoma) (Fig 2B). These *SOS1* mutations in five melanoma patients (R552K, G434R/V, C441F, and M239K) were found in the absence of *BRAF* and *NRAS* mutations. Furthermore, visual inspection of the pan-cancer incidence of *SOS1* mutations suggested a modest hotspot of 10 N233Y/I and 4 R552K/S mutations (Fig 3 and 3B). Mutations in *SOS1* are thought to activate Ras signaling [23,24], but while previous work has demonstrated the activation effect of M269R and E846K mutations (amongst others), G434R, N233Y, and R552S mutations have not been examined. To further validate our computational approach, we sought to experimentally validate the activation of the RAS-MAPK pathway by these *SOS1* variants. We transfected wild-type and mutant *SOS1* constructs into HEK293T cells and measured the effect on basal levels of phosphorylated ERK1/2. We observed a robust 2–6-fold activation depending upon the precise mutation. This is an effect size that is consistent with previous studies of Noonan syndrome [23,24] but more modest than BRAF V600E (Fig 3C). Thus, RAS-MAPK—activating mutants in *SOS1* occur in melanoma and other human tumors (Fig 3B), albeit with a more modest effect than current established drivers. Consistent with this more modest activity, SOS1 mutants alone were not sufficient to transform BaF3 cells while BRAF V600E was. Analyzing the spectrum of *SOS1* mutants across cancer indications suggests that these activating mutations can occur in a number of indications that we fail to call significant with our current cutoffs. As such, some true-positive overlap is not part of our 103 significant gene-cancer pairs. We suggest that this emphasizes the robustness and high stringency of the gene set that we do call significant. Thus, a minor subtype of Noonan syndrome and a newly discovered rare subtype of melanoma share activating variants in *SOS1*.

### *COL3A1* is a significantly mutated extracellular matrix cancer gene

**Fig 4:**
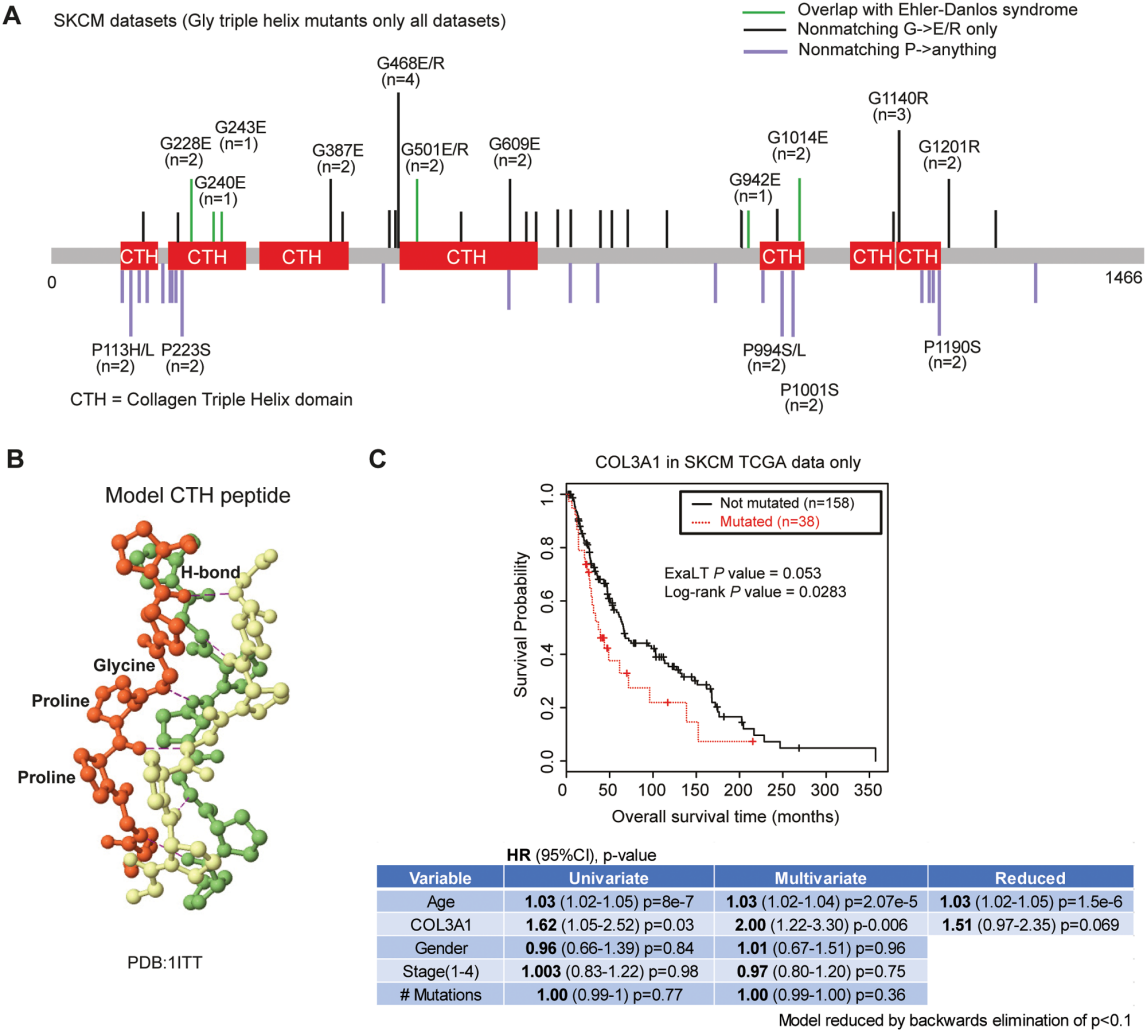
*COL3A1* mutations are associated with melanoma. **(A)** Reported mutations in *COL3A1* for all skin cutaneous melanoma studies. Mutations overlapping with Ehlers-Danlos syndrome are shown in green. Only glycine -> charged residue mutations (E/R) in conserved regions are shown for clarity. Examination of all melanoma mutations in *COL3A1* revealed numerous other glycine triple helix mutations that were distributed along the length of the protein, but are not documented to cause Ehlers-Danlos syndrome in the HUMSAVAR database (shown in black and labeled if N>2). Proline residues that also are important for the triple helix were also mutated (shown below in purple). **(B)** A model of collagen triple helix structure is shown. Hydrogen bonds stabilizing the triple helix conformation are shown. The tight-steric interactions of the proline residues and hydrogen bonding between the backbone nitrogen of the glycine residue and a carbonyl on an adjacent strand stabilize the triple helix conformation. Substitution of glycine with a bulkier residue directly disrupts these interactions. **C)** A Kaplan—Meier curve for all *COL3A1* mutations in the TCGA (others non-TCGA studies did not have survival data). *COL3A1* in SKCM was found to be modestly associated with decreased overall survival based upon log-rank, ExaLT and/or Cox regression. Cox regression outputs for univariate, multivariate, and reduced models with different effect sizes and *P* values are shown below the Kaplan-Meier curve. SKCM, skin cutaneous melanoma.

We next turned our focus to genes in pathways that are missed by previous approaches. One surprising finding was that mutations in the extracellular matrix gene *COL3A1* that are found in autosomal dominant Ehlers-Danlos syndrome patients, are significantly altered in patients with melanoma (Figs 4 and 2B). The mutations in *COL3A1* were glycine to glutamate substitutions at positions 228, 240, 501, 942, and 1014. These substitutions directly affect the ability of the Gly-X-Y triple helix motif to adopt the collagen triple helix fold (Fig 4A). Glycine is absolutely essential for triple-helix structure. In Ehlers-Danlos syndrome, *COL3A1* glycine mutations in the collagen triple helix are known to be pathogenic by a variety of molecular mechanisms that include impaired secretion, aberrant assembly, and lower stability [25,26]. However, they all share the consequence of creating tissues that lack fully functional *COL3A1*. Furthermore, G->E/R substitutions have similar structural effects that are agnostic to the exact location along the protein. Interestingly, our pathogenesis filter identifies recurring hits that are synchronous with the Gly-X-Y motif in the triple helix. This presents a distinct and novel form of “hotspot” that can only be detected in a filter like ours.

In Ehlers-Danlos syndrome, mutations in *COL3A1* result in an increased likelihood of the rupturing of vascular tissues [27]. An interesting hypothesis is that these mutations could be enhancers of metastasis. Interestingly, two experimental studies lend circumstantial support to this hypothesis. It has recently been shown that 4T1 tumor cells implanted into COL3A1 heterozygous mice (a COL3A1^+/-^ mouse is a haplo-insufficient mouse model for Ehlers-Danlos syndrome) grow and metastasize more aggressively [28]. While this demonstrates a cell non-autonomous functional effect of the loss of *COL3A1*, another recent study showed that *COL3A1* is secreted by cancer cells into the extracellular matrix at a high level in two distinct human cell lines [29]. These studies suggest that the loss of function of *COL3A1* in the extracellular matrix can have direct effects on tumor progression and that *COL3A1* can be secreted in a cell-autonomous fashion in experimental tumor models. Mutations in *COL3A1* are also associated with a decrease in overall survival (log-rank *P* value 0.028, ExaLT *P* value 0.053, and Cox *P* values that ranged 0.006–0.069 depending upon the covariates included (COL3A1 (N = 38), WT (N = 158)). While the number of *COL3A1* mutant tumors is small, the decreased survival is modestly significant across a variety of statistical analyses. Repetition in independent cohorts will be important to determine the reproducibility of these observed survival differences. Thus, a devastating and rare genetic disease (Ehlers-Danlos Syndrome), and a subset of melanoma patients share pathogenic variants in *COL3A1*. This suggests that there may be a larger unmet medical need for therapies that can ameliorate the effects of *COL3A1* mutations than was previously known.

### Familial cancer genetics identifies rare and targetable alterations in sporadic lung cancer

We also found the first evidence for significant variants in R24 of *CDK4* in lung adenocarcinoma. R24 mutations have been previously described only in familial and sporadic melanoma [30]. These R24L variants reside in the CDKN2A binding site, and when combined with the nearby K22M mutants across all melanoma datasets are mutually exclusive with CDKN2A alterations (*P* value < 0.001, Fisher exact test (CDK4 N = 7, CDKN2A N = 108, None N = 275). Thus, we show that set of mutations previously only known to exist in familial and sporadic melanoma, exist in lung cancer, and are mutually exclusive with established alterations in the same pathway. Interestingly, potent inhibitors of CDK4/6 are already approved in breast cancer [31].

### Kinase-activating mutations in *ACVR1* are found in uterine corpus endometrial cancer and increase the potential unmet medical need for anti-*ACVR1* therapy

**Fig 5:**
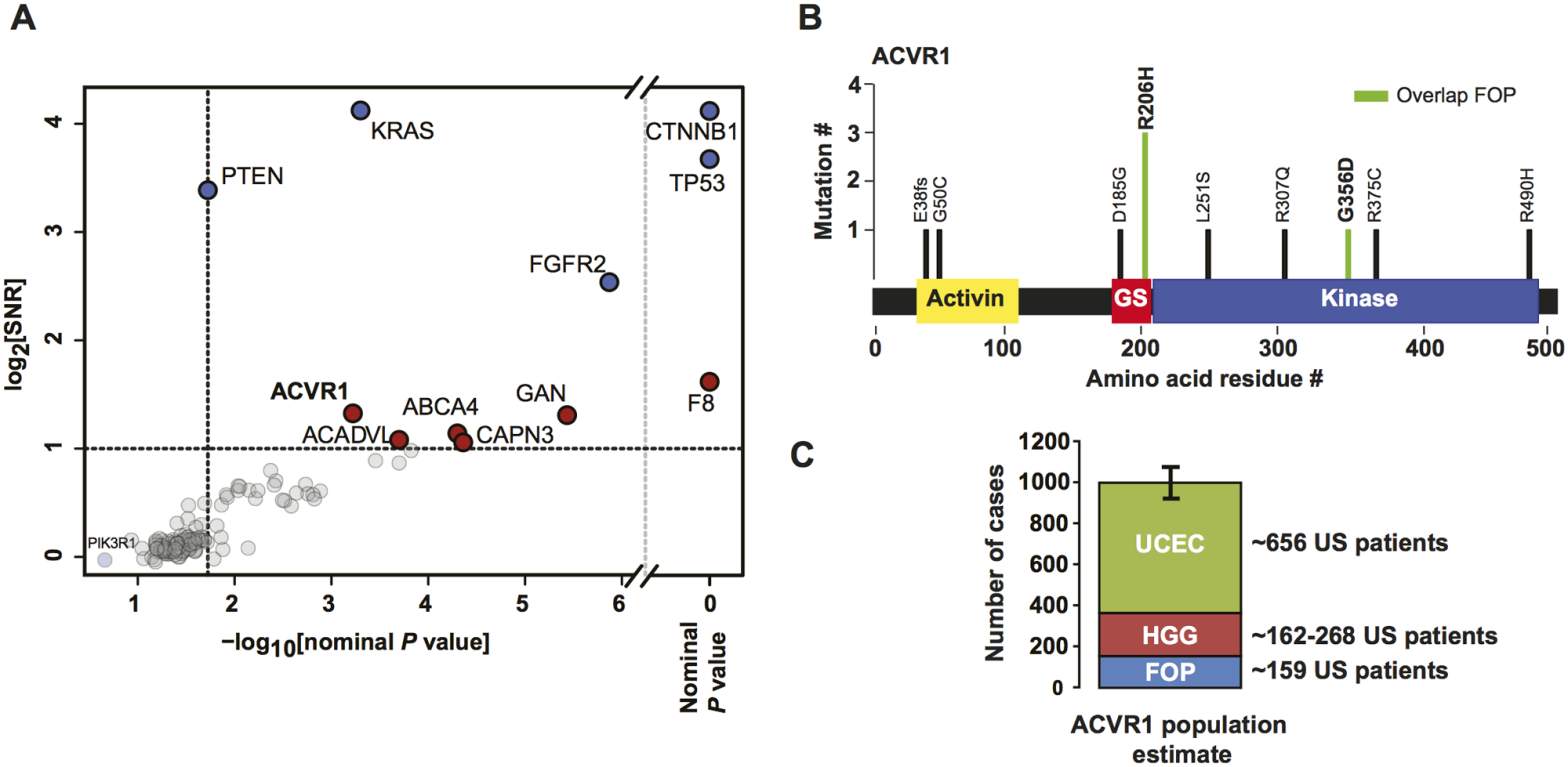
*ACVR1* mutations in endometrial cancer. **(A)** All overlapping residues between the two databases are scored for signal-to-noise ratios and nominal *P* values. Blue dots represent the hits that are also significant by MutSigCV. The red dots are new associations found in this study. The distribution of *P* values associated with the expressed genes have a long tail. **(B)** *ACVR1* mutations in endometrial cancer. Overlap with FOP is denoted in green. GS is the glycine serine rich domain. **(C)** An estimate of the cumulative incidence of *ACVR1*-mutant diseases. Error bars are based on the difference in *ACVR1* frequency estimates in pediatric high-grade glioma and represent min/max estimates of incidence. FOP, fibrodysplasia ossificans progressive; HGG, high-grade glioma; UCEC, uterine corpus endometrial cancer.

Finally, we identified a significant overlap between the kinase-activating mutations in *ACVR1* that cause the debilitating genetic disorder (FOP) and cancer of the endometrium (Fig 5A). *ACVR1* mutations in endometrial cancer overlapped with FOP at two distinct positions (Fig 5B), and were significantly associated with variants in the PTEN-AKT-mTOR pathway (*P* value combined by Fisher’smethod 0.0001) (S8 Fig). *ACVR1* mutations occurred in 3.3% of endometrial cancers in the TCGA dataset, and about half of those variants overlapped with FOP. Four publications recently found the same mutations in high-grade pediatric astrocytomas and gliomas [10,18–20]. Epidemiologically, FOP occurs at an incidence of 1 in 2 million, and pediatric high-grade gliomas occur in less than 1 in 100000 children. Collectively, these two indications may represent an unmet need for anti-*ACVR1* therapy of less than 500 patients in the US patient population. Inclusion of patients with endometrial cancer more than doubles the potential patient population (Fig 5C).

Biologically, the existence of *ACVR1* mutations in endometrial tumors is especially interesting. Bone morphogenetic protein 2 (BMP2)–ACVR1 signaling was recently found to be necessary for embryo implantation and decidualization (a process that requires invasion, proliferation, and angiogenesis) in knockout mouse studies of the uterus [32,33]. Loss of BMP2 in the uterus also leads to a down-regulation in mTOR at the site of implantation [33]. This suggests that the presence of these mutations in endometrial tumors, and their association with the mTOR pathway, is consistent with the dysregulation of a known physiological role of ACVR1 signaling in endometrial tissue.

We have developed a methodology to predict significant overlap between variants in inherited disease and cancer genetic databases. Using this method, we have added new cancer associated genes, substantially expanded the therapeutically actionable population of patients harboring activating mutations in *ACVR1*, and identified the first evidence of significant mutations in *CDK4* inlung cancer and in *SOS1* inmelanoma. We found that a surprisingly large number of inherited disease variants will overlap with mutations in cancer exomes based upon chance alone. This suggests that merely observing the presence of a variant that is known to have another effect in a cancer genome is not sufficient to predict its cancer relevance in patients. Our analyses will enable anyone with a database of amino acid variants from diverse methodologies (e.g., mutagenesis screens or biochemical analyses) to investigate whether the variants in their lists are significantly altered in cancer. Because our algorithm explicitly analyzes overlap, it will always be limited by the existence of overlapping variants in different databases. This means that our method can only add power to other cancer significance callers, it is not meant to be a replacement for methods like MutSigCV.

However, our method is generalizable to any database of variants. Thus, any group that discovers a specific variant in any context can use our method to ask whether that variant occurs more often than would be expected in the cancer genome. This work will add statistical rigor to studies that identify functional impacts of an amino acid residue and observe that variant in cancer.

## Supporting Information

S1 Fig Background tumor-specific proportionality coefficient estimation using various RNA-seq cutoffs. We estimated the background proportionality coefficients using a subset or all of the mutation data per tumor type. This estimation is fairly stable, i.e., it is independent of the RNA-seq cut-offs we used.
(TIFF)

S2 Fig Empirical cumulative distribution function of *P* values for all overlapping genes. **A.** Empirical cumulative distribution function (eCDF) illustrates a much longer tail for small *P* values for the expressed genes compared with nonexpressed genes. Expressed genes are in red and nonexpressed genes are in blue. **B.** Three different read number cutoffs were examined. A Venn-diagram depicts the number of overlapping hits when 1, 5 or 10 reads were used to determine whether a gene was or was not expressed.
(TIFF)

S3 Fig Comparison of our results with a co-morbidity based approach. A Venn diagram comparing the performance of our methodology to that of Melamed et al 2015 [15]
(TIFF)

S4 Fig Comparison of the effect of different cancer exome datasets on the number of hits discovered. **A.** A schematic of our data acquisition and filtering pipeline. Variants are removed from top to bottom. The datasets at the bottom of the filtering pipeline used as inputs to our algorithm to identify significant overlap. In addition, we also performed filtering to focus only on TCGA or in overlaying with PanCancer12 [1] as indicated by the dotted box, with resulting filtered dataset used for comparison, as shown in B and C. **B**. Venn-diagram depicting the influence of removing nonTCGA variants on number of significant hits identified. Indications LIHC, MM, and SCLC were excluded as variants were not from TCGA source (at the time of acquisition from cBioPortal). **C.** Venn-diagram depicting the number and overlap of significant hits when we used the standardized input of Kandoth et al. 2013. Indications ACC, KICH, and UCS (in addition to LIHC, MM, and SCLC) were excluded as these are not in TCGA. **D.** Hits added versus variants added for the comparisons in B and C. Line drawn based on linear regression on the data, with shading depicting 95% confidence interval.
(TIFF)

S5 Fig Comparison of inherited disease input datasets. **A.** A schematic of the download and filtering of the HUMSAVAR database. Variants are removed from top to bottom. **B.** A schematic of the download and filtering of the ClinVar database. Variants are removed from top to bottom. **C.** A comparison of the outputs of the algorithm when using either HUMSAVAR or ClinVar as the input dataset. There is substantial overlap in results.
(TIFF)

S6 Fig Effects of statistical filter on hits identification. **(A, B)** Statistics-naïve exact match overlay between the inherited diseases and TCGA datasets. There are an overwhelming number of genes with exact mutational matches, suggesting the potential for a large number of false-positives and a need for a more rigorous statistical filter. Known potential false-positives such as RYR2 (highlighted in red) are on this list. **(C, D)** Our statistical filter (see methods for details of statistical model) generates parsimonious statistically significant list of hits, with the disappearance of RYR2 and enrichment of other hits such as *FGFR2* and *FGFR3* (highlighted in orange).
(TIFF)

S7 Fig Statistical analyses of results From synthetic data. **(A)** Nominal *P* values do not correlate with protein length. **(B)** Nominal *P* value is minimally negatively correlated with mutation burden (i.e., number of residues mutated over protein length). This, in fact, overpenalizes as a function of mutation burden, and as such, improves sensitivity at the expense of specificity. *P* value was determined based upon a proportionality coefficient (*γ*) of 10.
(TIFF)

S8 Fig Associations of *ACVR1* with genes in PTEN-AKT-MTOR pathway. A matrix of *P* values (Fisher exact test) for genes within the PTEN-AKT-mTOR pathway, and between the pathway and *ACVR1*.
(TIFF)

S1 Text Supplementary results and discussion section. (DOCX)

S1 Data Extensive statistics on variant numbers and filters. (XLSX)
