# Supplementary figures and images for: Inherited Disease Genetics Improves the Identification of Cancer-Associated Genes
Source: PLoS Genet. 2016 Jun 15;12(6):e1006081. doi: 10.1371/journal.pgen.1006081 (PMC4909226; doi:10.1371/journal.pgen.1006081)

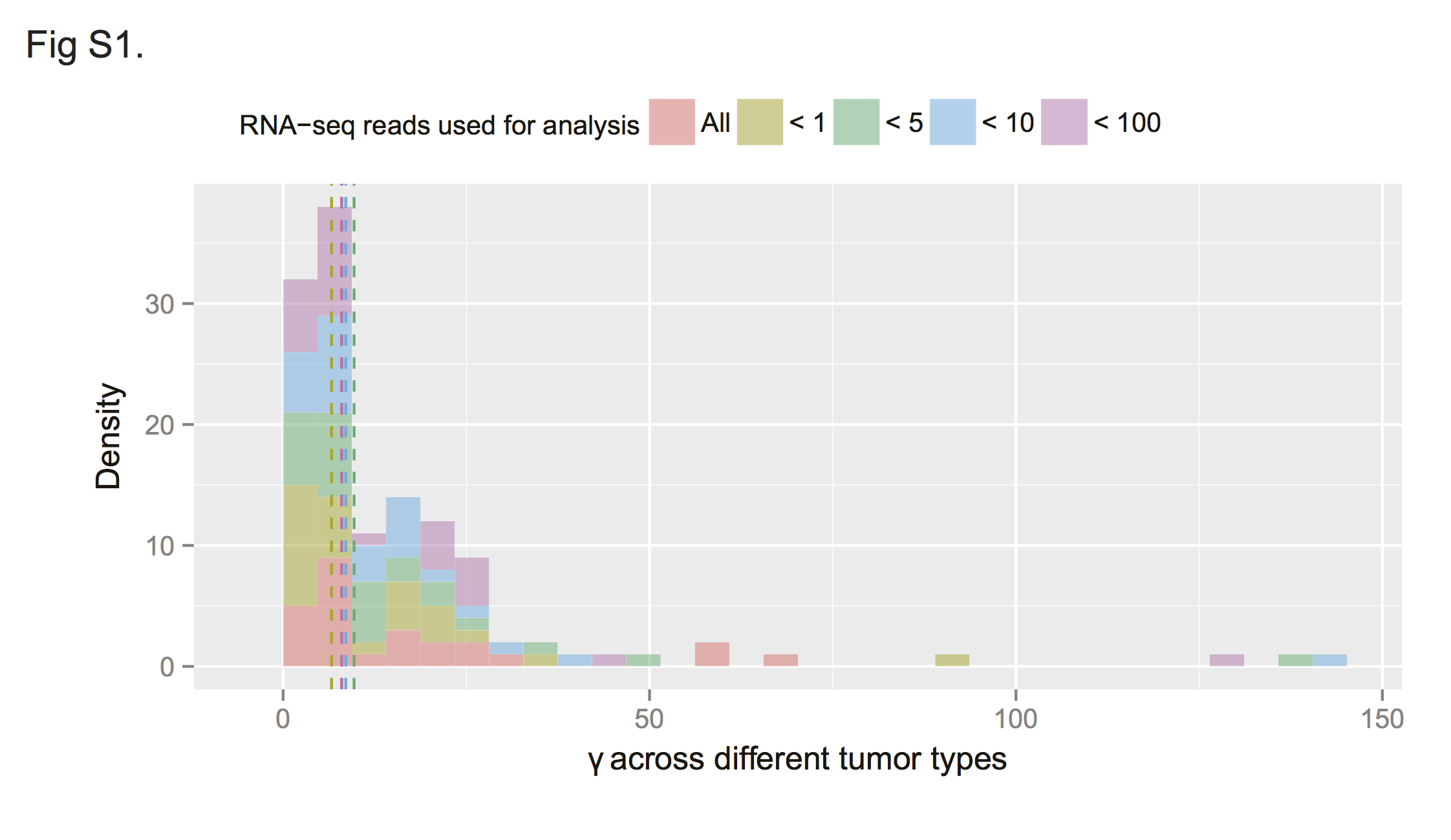

Supplement: S1 Fig — We estimated the background proportionality coefficients using a subset or all of the mutation data per tumor type. This estimation is fairly stable, i.e., it is independent of the RNA-seq cut-offs we used. (TIFF) [file pgen.1006081.s001.tiff]

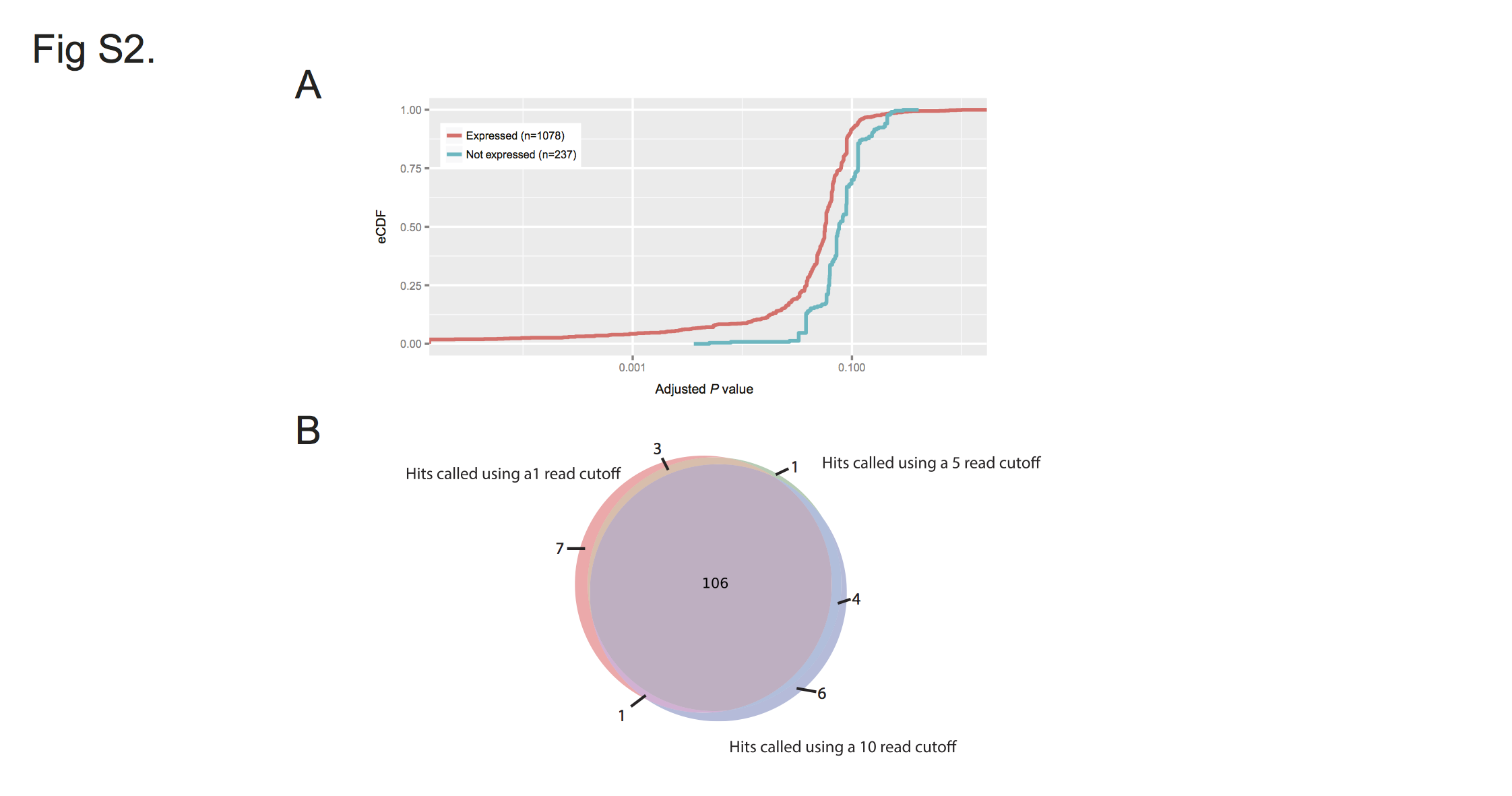

Supplement: S2 Fig — A. Empirical cumulative distribution function (eCDF) illustrates a much longer tail for small P values for the expressed genes compared with nonexpressed genes. Expressed genes are in red and nonexpressed genes are in blue. B. Three different read number cutoffs were examined. A Venn-diagram depicts the number of overlapping hits when 1, 5 or 10 reads were used to determine whether a gene was or was not expressed. (TIFF) [file pgen.1006081.s002.tiff]

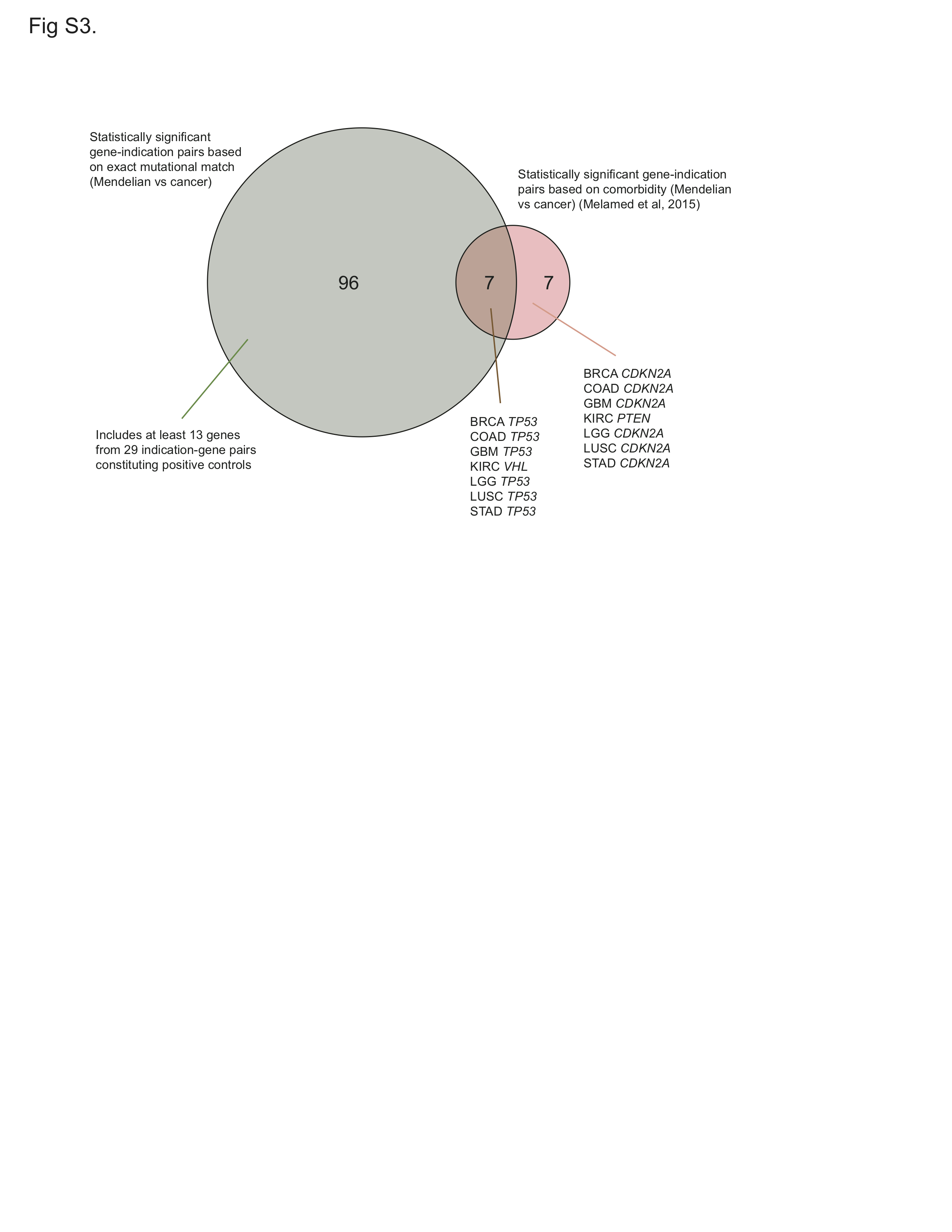

Supplement: S3 Fig — A Venn diagram comparing the performance of our methodology to that of Melamed et al 2015 [15] (TIFF) [file pgen.1006081.s003.tiff]

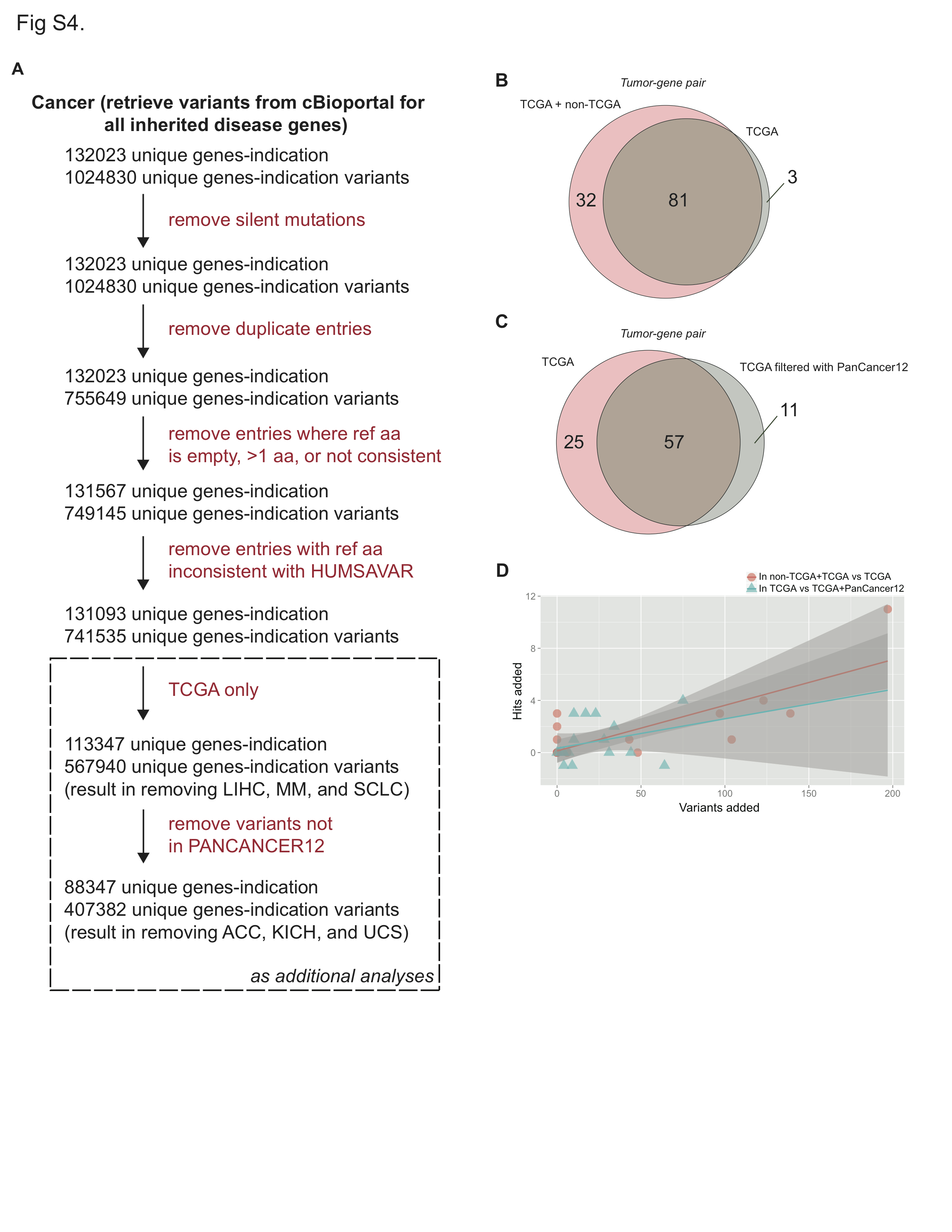

Supplement: S4 Fig — A. A schematic of our data acquisition and filtering pipeline. Variants are removed from top to bottom. The datasets at the bottom of the filtering pipeline used as inputs to our algorithm to identify significant overlap. In addition, we also performed filtering to focus only on TCGA or in overlaying with PanCancer12 [1] as indicated by the dotted box, with resulting filtered dataset used for comparison, as shown in B and C. B. Venn-diagram depicting the influence of removing nonTCGA variants on number of significant hits identified. Indications LIHC, MM, and SCLC were excluded as variants were not from TCGA source (at the time of acquisition from cBioPortal). C. Venn-diagram depicting the number and overlap of significant hits when we used the standardized input of Kandoth et al. 2013. Indications ACC, KICH, and UCS (in addition to LIHC, MM, and SCLC) were excluded as these are not in TCGA. D. Hits added versus variants added for the comparisons in B and C. Line drawn based on linear regression on the data, with shading depicting 95% confidence interval. (TIFF) [file pgen.1006081.s004.tiff]

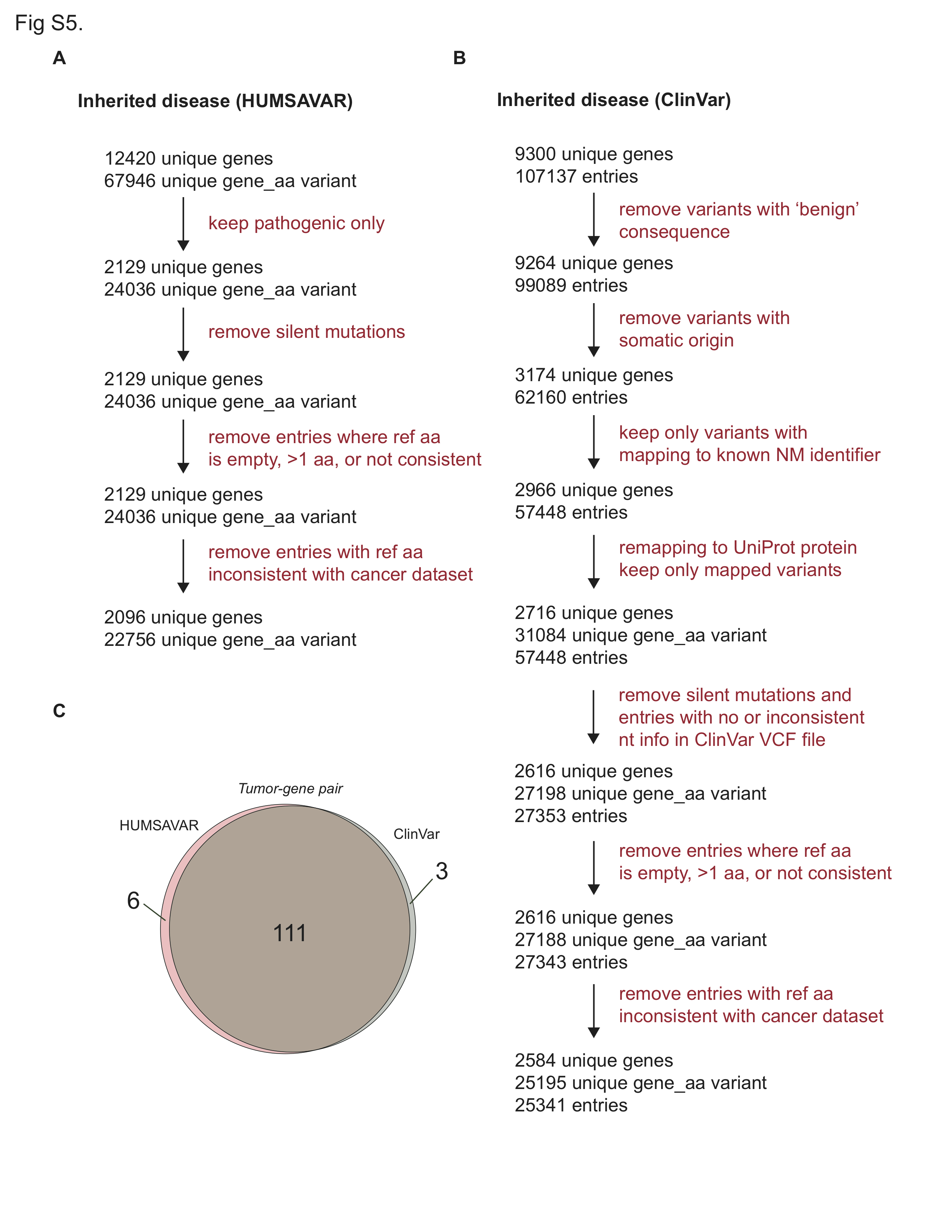

Supplement: S5 Fig — A. A schematic of the download and filtering of the HUMSAVAR database. Variants are removed from top to bottom. B. A schematic of the download and filtering of the ClinVar database. Variants are removed from top to bottom. C. A comparison of the outputs of the algorithm when using either HUMSAVAR or ClinVar as the input dataset. There is substantial overlap in results. (TIFF) [file pgen.1006081.s005.tiff]

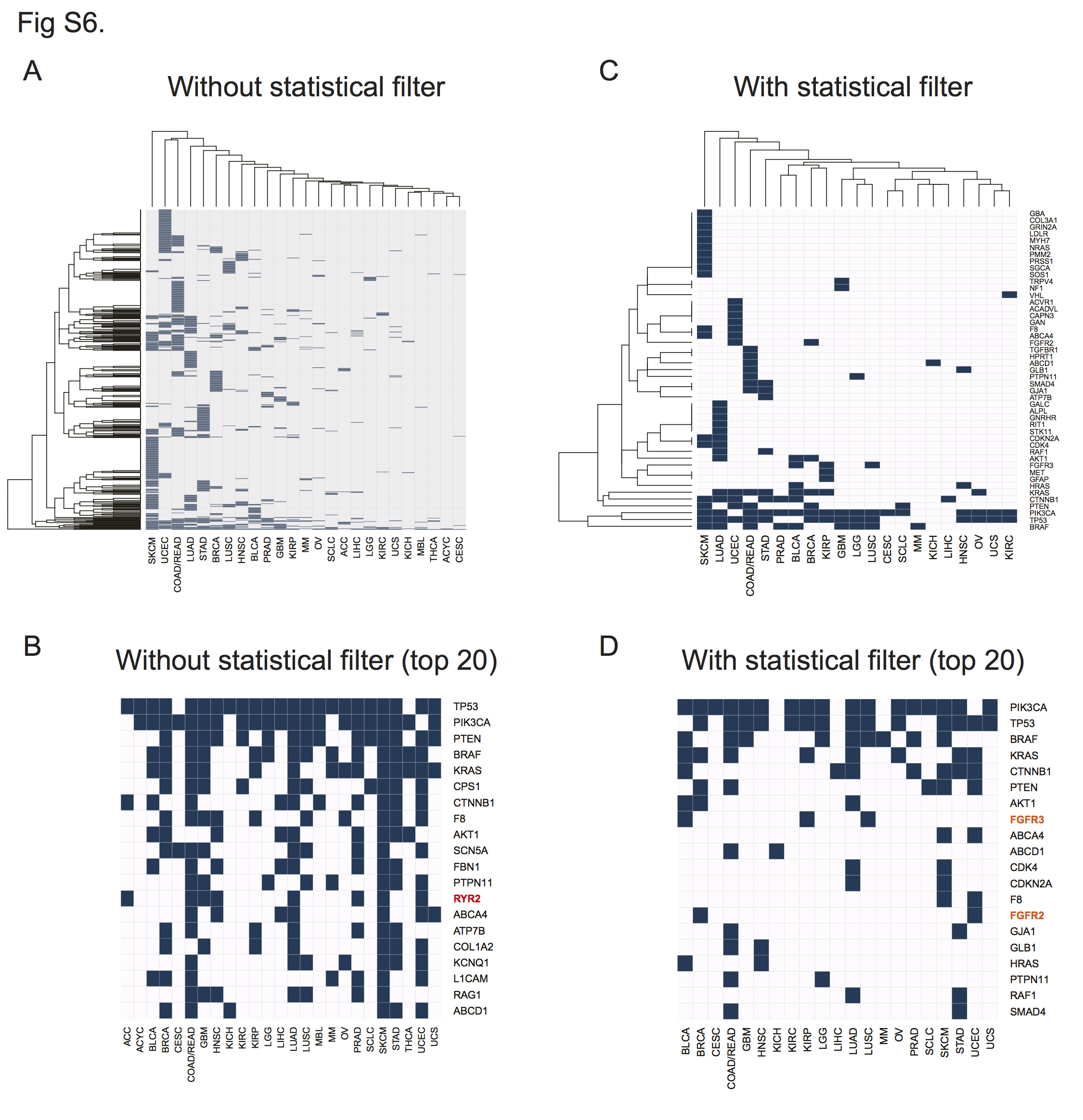

Supplement: S6 Fig — (A, B) Statistics-naïve exact match overlay between the inherited diseases and TCGA datasets. There are an overwhelming number of genes with exact mutational matches, suggesting the potential for a large number of false-positives and a need for a more rigorous statistical filter. Known potential false-positives such as RYR2 (highlighted in red) are on this list. (C, D) Our statistical filter (see methods for details of statistical model) generates parsimonious statistically significant list of hits, with the disappearance of RYR2 and enrichment of other hits such as FGFR2 and FGFR3 (highlighted in orange). (TIFF) [file pgen.1006081.s006.tiff]

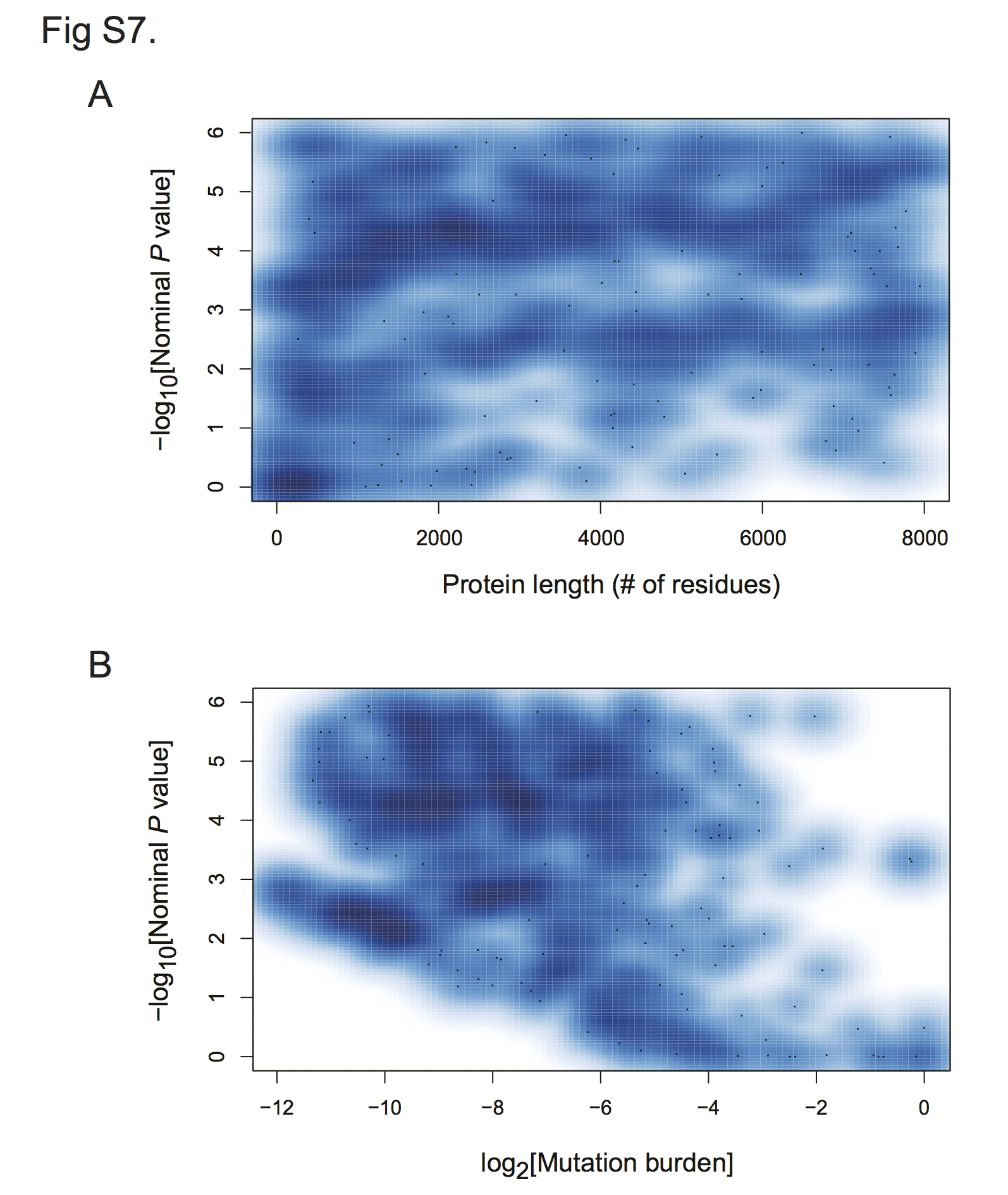

Supplement: S7 Fig — (A) Nominal P values do not correlate with protein length. (B) Nominal P value is minimally negatively correlated with mutation burden (i.e., number of residues mutated over protein length). This, in fact, overpenalizes as a function of mutation burden, and as such, improves sensitivity at the expense of specificity. P value was determined based upon a proportionality coefficient (γ) of 10. (TIFF) [file pgen.1006081.s007.tiff]

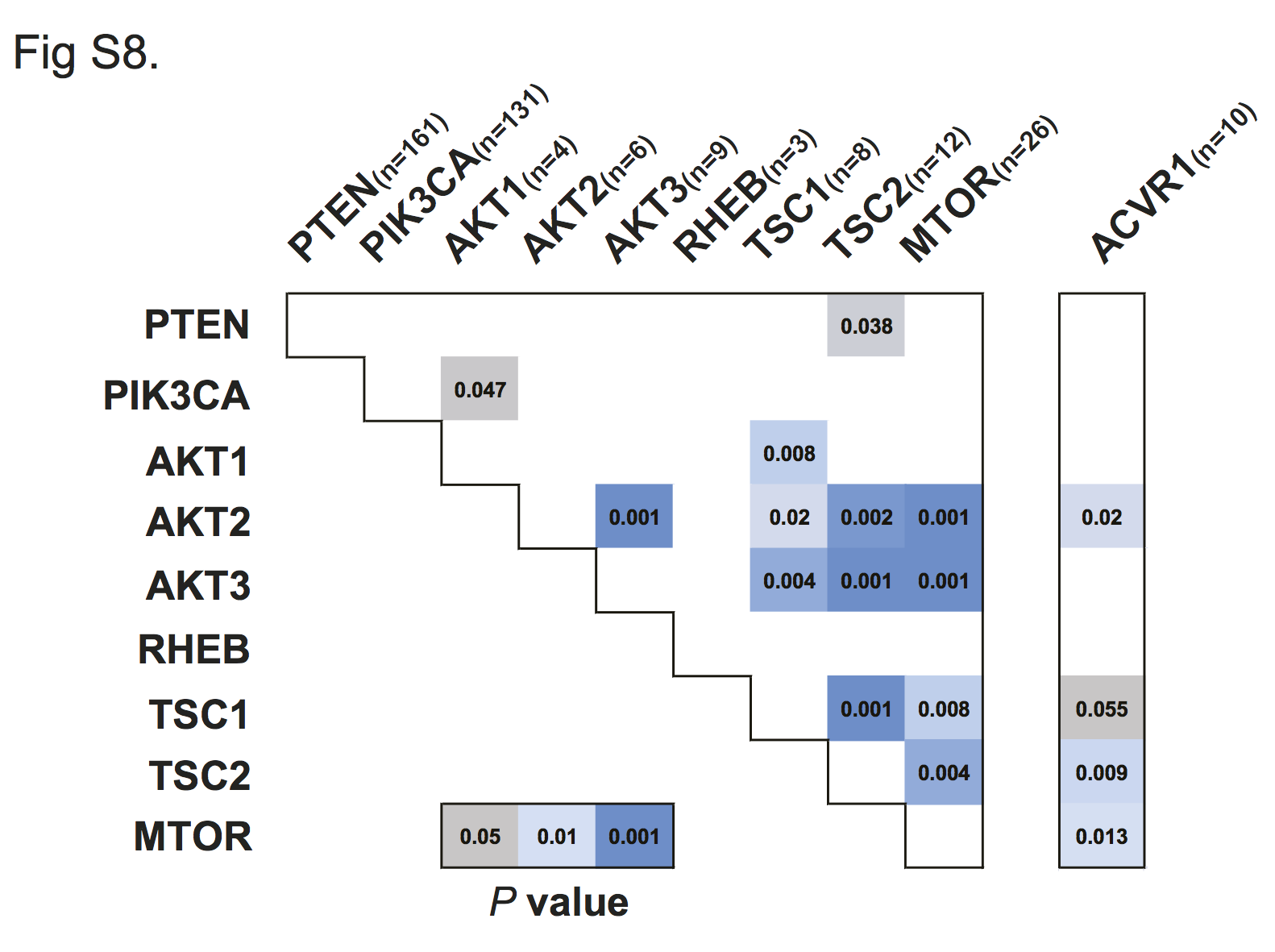

Supplement: S8 Fig — A matrix of P values (Fisher exact test) for genes within the PTEN-AKT-mTOR pathway, and between the pathway and ACVR1. (TIFF) [file pgen.1006081.s008.tiff]
