# Supplementary material for: Inherited Disease Genetics Improves the Identification of Cancer-Associated Genes
Source: PLoS Genet. 2016 Jun 15;12(6):e1006081. doi: 10.1371/journal.pgen.1006081 (PMC4909226; doi:10.1371/journal.pgen.1006081)
Supplement: S1 Text — (DOCX) [file pgen.1006081.s009.docx]

**Supplemental Results and Discussion for *Inherited Disease Genetics Improves the Identification of Cancer-Associated Genes***

**Boyang Zhao and Justin R. Pritchard**

*Comparison to the co-morbidity based approach of Melamed et al.*

We identified 103 inherited disease gene–cancer pairs (of the cancer indications also examined in the Melamed et al study [1]). Of these, seven were identified in the Melamed et al [1] study using the comorbidity based approach with gene intersection. Of the pairs that we uniquely identified, 29 pairs were in 13 genes with experimental validation of their driver status and were called significant by MutSigCV, thus constituting strong positive controls. These included *TP53*, *PIK3CA*, *CTNNB1*, *KRAS*, *PTEN*, *BRAF*, *CDKN2A*, *NRAS*, *SMAD4*, *HRAS*, *NF1*, *RIT1*, and *STK11*. Six out of seven Melamed et al comorbidity gene-disease pairs that we consistently failed to identify were loss-of-function mutations in *CDKN2A*—five of which also were not identified by MutSigCV (*possibly due to CNV alteration)* (Fig S3). Finally, of the gene-disease pairs that we missed, but that MutSigCV called significant, 78% lack an exact match in an inherited disease. Thus, our approach functions as expected; only adding statistical power when overlap in the variant databases exists and outperforms the previous approach of Melamed et al. This also highlights a key distinction of our approach versus MutSigCV. We do not aim to replace other significance callers, but to add significant signal to noise when independent biological data exist with resolution at the amino acid level.

*Comparison of algorithm performance across different input exome data sets and input inherited disease datasets.*

We start with the assumption that most complete aggregation of cancer exome data would combine both TCGA and non TCGA data sources from many different exome studies in the literature to increase the statistical power to identify new cancer drivers. This rationale led us to utilize the cBioPortal as the source of our exome data. However, there are significant heterogeneities in variant calling procedures in different cancer sequencing studies. Even within the TCGA, different working groups use different standards to tune the sensitivity and specificity for variant calls in distinct cancer indications.

In “ pan-cancer” studies it is important to consider the goals and methods of the study when deciding which filters/pipelines to utilize. Across cancer studies should pay attention to differences in variant calling pipelines when they compare across TCGA studies (i.e. examining differences in mutations rates/mechanisms/biases amongst indications) or doing statistical analyses on data compiled across all TCGA studies.

Even in these across cancer studies, there is a division in the literature between studies such as Kandoth et al. 2013[2] and Miller et al 2015[3] versus Lawrence et al. 2013[4]. Kandoth and Miller use filtered MAFs after TCGA curation, while Lawrence et al. utilizes the Broad institute’s firehose platform. Importantly, both sets of authors seek a different solution to the problem of bias across variant calling pipelines in the TCGA. MAFs incorporate a large amount of expert curation across multiple mutation callers to determine a high confidence set of variants. While utilizing a single pipeline homogenizes variation between studies, it introduces the bias of that single pipeline. This debate in the literature is especially relevant in studies like Kandoth and Lawrence, where the authors seek to compare mutation rates, and mutational mechanisms across different TCGA indications. Thus “across cancer types” comparisons require consideration of the different variant call standards in different TCGA working groups and other studies. Interestingly despite their difference in approaches, Kandoth and Lawrence reach very similar conclusions about mutation rates across indications.

Our study is a different type of “pan-cancer” study. We make no comparisons across cancer types. Our simulation based approach accounts for variant call differences by comparing to simulations run on data from that same cancer study. Nonetheless, we examined how different inputs (standardized in different ways) changed our algorithm outputs. We utilized our full data set which included all TCGA and Non-TCGA studies on cBioPortal. This included 7362 exomes and 741535 variants. We removed the non TCGA data and were left with 567940 variants. Finally, we used the Pan-Cancer 12 standardized dataset from Kandoth et al. 2013, this dataset is all TCGA data as of the earlier data lock of the Kandoth study. Therefore the Kandoth pan-cancer 12 is a subset of the TCGA dataset that we have used and filtering using this dataset resulted in 407382 variants. Details of variants, filters, datasets and hits can be found in S4 Fig A, S1 Data. We used each of these datasets as input to our algorithm and applied the same variant call and expression filters. TCGA and non-TCGA are 70% concordant in hit calls while the Kandoth pancancer 12 is 61% concordant with our full TCGA dataset (S4 Fig B,C). In both comparisons, the larger dataset (containing more variants and variant matches, Table S1) has the expected behavior of calling more hits. To examine the stability of our algorithm across the different datasets, we looked at the rate of hits added per additional matching variant in the dataset. Importantly the rate of hits per match does not vary significantly across input types (~2 hits per 50 matching variants added vs ~1.5 hits per 50 matching variants added FS4 Fig D). Thus, we believe that our algorithm statistics are stable across different variant call standards, and that the observed differences in hit calls are consistent with the added statistical power that is inherent in larger sample sizes. Consistent with this added power hypothesis is Fig 3B, SOS1 (which we experimentally validate to be RAS/MAPK activating) is differentially called in non-TCGA versus TCGA inputs. This is because 4/5 SKCM variants were found in Non-TCGA studies. However, Noonan syndrome overlap was found 19 times across 12 different cancer exome study pipelines. Thus the true positive overlap of SOS1 is only called with the added statistical power of additional study exomes, even though overlapping variants in Noonan syndrome residues are recurrently called across studies. Different hits that are called from different inputs can be found in our GitHub collection (./targetID/siglist/).

We suggest that different variant call pipelines/standards amongst different sequencing studies are not a major confounding factor in our analysis, but we believe it does limit our sensitivity. We make no comparisons across different cancer types that utilize different curation techniques. An argument that would be innapropriate is, “skin cancer is more like Noonan syndrome (a genetic disease) than kidney cancer”. We do not attempt to make these statements as we do not believe this is an interesting comparison. It is important to note that while our algorithm’s assessment of significant overlap appears quantitatively stable across different inputs, it cannot call driver overlap significant if an overlapping variant is not identified. Thus while our method is insensitive to false positives caused by different variant pipelines, it will be sensitive to false negatives.

Next we examined different inherited disease datasets that include differential levels of curation, input sources, and standards for calling pathogenicity. HUMSAVAR draws from OMIM and is curated by UNIPROT. ClinVar draws from OMIM, dbSNP, Gene Reviews and user updates. While the majority of pathogenic variants in both databases have OMIM accession numbers (including 99.5% of HUMSAVAR “disease associated”, and 70% of ClinVar variants that are not “benign”). In S5 Fig A,B we provide in depth variant statistics for our processing pipeline and filters. We next used both filtered datasets as inputs to our algorithm. We observed a high concordance in significant overlap between these two inherited disease databases. 111/116 HUMSAVAR hits were called with ClinVar, and 111 of 114 ClinVar hits were called with HUMSAVAR. This suggests that in spite of the differences in database provenance, our algorithm finds a similar set of significant hits (S5 Fig C).

**References**

1. Melamed RD, Emmett KJ, Madubata C, Rzhetsky A, Rabadan R. Genetic similarity between cancers and comorbid Mendelian diseases identifies candidate driver genes. Nat Commun. Nature Publishing Group; 2015;6: 7033. doi:10.1038/ncomms8033

2. Cyriac Kandoth, Michael D. McLellan, Fabio Vandin, Kai Ye BN and CL. Mutational landscape and significance across 12 major cancer types. Nature. 2013;503: 333–339. doi:10.1007/s13398-014-0173-7.2

3. Miller ML, Reznik E, Gauthier NP, Aksoy BA, Korkut A, Gao J, et al. Pan-Cancer Analysis of Mutation Hotspots in Protein Domains. Cell Syst. 2015;1: 197–209. doi:10.1016/j.cels.2015.08.014

4. Lawrence MS, Stojanov P, Polak P, Kryukov G V, Cibulskis K, Sivachenko A, et al. Mutational heterogeneity in cancer and the search for new cancer-associated genes. Nature. Nature Publishing Group, a division of Macmillan Publishers Limited. All Rights Reserved.; 2013;499: 214–218.
